# Global sagittal alignment after surgery of right thoracic idiopathic scoliosis in adolescents and adults with and without thoracic hypokyphosis

**DOI:** 10.1038/s41598-021-85782-6

**Published:** 2021-03-18

**Authors:** Kwong Hang Yeung, Gene Chi Wai Man, Wafa Skalli, Zongshan Hu, Vivian Wing Yin Hung, Alec Lik Hang Hung, Tsz Ping Lam, Bobby Kin Wah Ng, Jack Chun Yiu Cheng, Claudio Vergari, Winnie Chiu Wing Chu

**Affiliations:** 1 Department of Imaging and Interventional Radiology, Faculty of Medicine, The Prince of Wales Hospital, The Chinese University of Hong Kong, Shatin, Hong Kong SAR China; 2 SH Ho Scoliosis Research Laboratory, Department of Orthopaedics and Traumatology, Faculty of Medicine, The Prince of Wales Hospital, The Chinese University of Hong Kong, Shatin, Hong Kong SAR China; 3 Arts et Métiers Institute of Technology, Université Sorbonne Paris Nord, IBHGC - Institut de Biomécanique Humaine Georges Charpak, HESAM Université, Arts et Metiers ParisTech, 151, boulevard de l’hopital, 75013 Paris, France

**Keywords:** Genetics, Molecular biology, Biomarkers, Medical research, Risk factors

## Abstract

This study aimed to characterize global sagittal alignment in adolescent idiopathic scoliosis (AIS) with normal kyphosis (NTK, kyphosis > 10°) and with thoracic hypokyphosis (THK, kyphosis < 10°), before and after posterior spinal fusion, and compare them with asymptomatic controls. 27 AIS girls and young adults with right thoracic curves were included (seventeen with age ≤ 18 years, then age > 21). Biplanar radiographies were acquired at baseline, immediate post-operatively, 1-year and 2-year follow-up, and 3D reconstruction of the spine and pelvis was performed. NTK and THK showed different global sagittal alignment, as well as differences compared to controls. AIS with THK at baseline had higher SVA/SFD (2.0 ± 2.9 vs − 0.4 ± 1.9; *P* < 0.05) and OD-HA (0.2 ± 1.4° vs − 1.3 ± 1.6°; *P* < 0.05) than controls, indicating that THK had compensated balance with unusual forward leaning posture. Immediately post-operation, SVA/SFD remained high (1.3 ± 3.0) while OD-HA reversed (− 1.2 ± 1.7°), indicating that THK patients had found partially compensated balance. After 2-yeas, both SVA/SFD (− 1.3 ± 2.1) and OD-HA (− 1.4 ± 0.9°) were normalized. The changes in global sagittal alignment and mechanism of balance are different in AIS with or without THK. As the head plays a critical role on balance during immediate and delayed post-operation, OD-HA can be complementary parameter for assessing global balance during post-operative follow-up of AIS patients with THK.

## Introduction

Adolescent idiopathic scoliosis (AIS) is a three-dimensional deformity of the spine for which the cause has not yet been identified^1^. Current surgical treatment for AIS involves multi-segmental posterior pedicle screw instrumentation and spinal fusion, which can achieve considerable correction in the coronal plane with limited loss of correction over time^2^. Most reports on the surgical correction mainly focused on the coronal Cobb angle, with relatively little emphasis on the changes of sagittal profile. Some studies have demonstrated that an insufficient surgical correction of hypokyphosis in severe AIS can lead to sagittal malalignment and accelerated degeneration of the distal unfused vertebral segments^3,4^.

Indeed, keeping an ideal standing posture is important to ensure the least amount of energy expenditure to prevent muscle fatigue and vertebral strain^5^. Several authors studied the sagittal plane in AIS, and in particular the relationship between the thoracolumbar spine and the pelvis^6^. In some cases, cervical spine was included^7^. When considering the changes of the sagittal alignment after surgery, Park et al. followed-up 93 patients for 2 years, for instance, but they limited their analysis to the thoracolumbar spine^4^, while other studies included the cervical spine. Still, the position of the head is often neglected in these analyses. With a mass of 4–5 kg, the head engages multiple vertebral segments to obtain an economic balance, and the neuromuscular system aims at keeping the gaze horizontal and the head within a narrow cone above the pelvis^8^. Including the head position in the analysis of global sagittal alignment allows to analyze the postoperative changes of alignment in terms of compensation mechanisms^9^.

In this study, we aimed to analyze global sagittal alignment in AIS, with a focus on the head position, according to thoracic hypokyphosis after posterior spinal fusion, and relative to asymptomatic controls, with the hypothesis that such analysis might improve the understanding of sagittal compensating mechanisms from head to pelvis during early and later post-operative periods.

## Results

### Subjects’ characteristics

**Figure 1:**
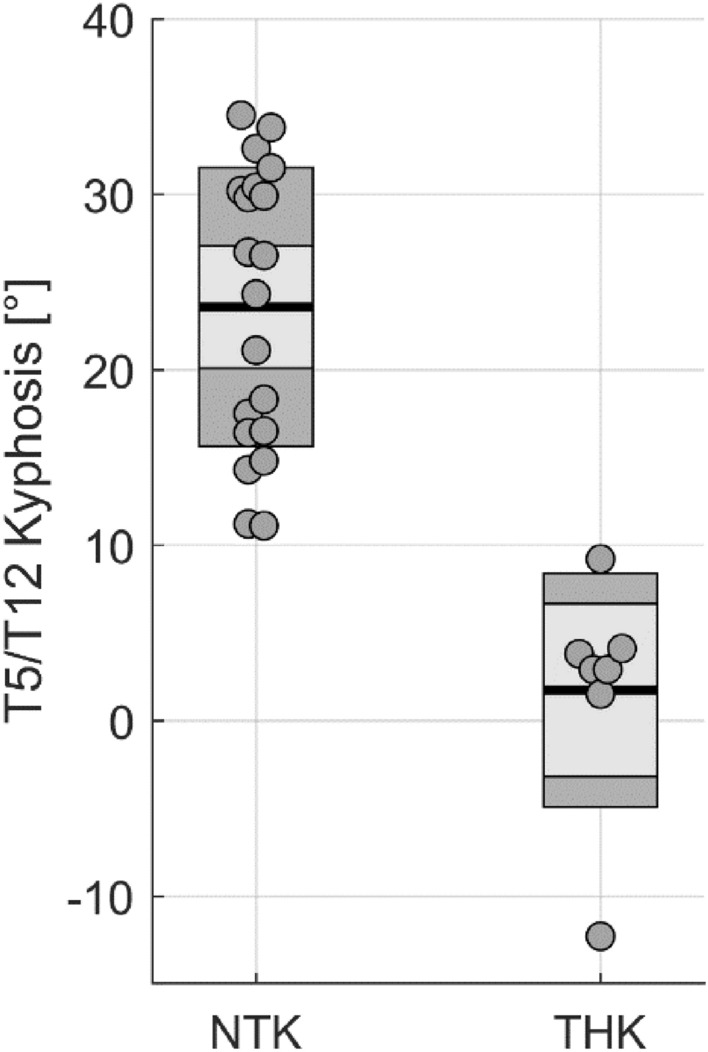
Distribution of T5/T12 kyphosis in patients with normal thoracic kyphosis (NTK) and hypokyphosis (THK).

Twenty-seven patients with AIS (female; mean age: 18.1 ± 4.6yrs, ranging between 12 and 31 years old) and thirty-six asymptomatic controls (female; mean age: 25.1 ± 2.7yrs) were recruited (Table 1). Seventeen patients were 18 years old or younger at time of surgery, while 10 were older. Most patients were presented with Lenke type 1 or 2 curvatures (24 out of 27). Mean Cobb angle at pre-operative and at immediate post-operative were 65.4 ± 11.9° and 21.5 ± 8.2°, respectively (*P* < 0.001), indicating a correction rate of 68 ± 9.9%. Based on Lenke classification^10^, seven AIS patients had thoracic hypokyphosis (THK; T5-T12 kyphosis = 1.7 ± 6.6^°^) and twenty AIS patients had normal thoracic kyphosis (NTK; T5-T12 kyphosis = 23.6 ± 7.9°, *P* < 0.001). Only one THK patient was older than 18 (21 years old, T5-T12 kyphosis of 3.8°); therefore, no analysis was carried out on the older THK patients’ sub-group. Figure 1 shows the distribution of T5/T12 kyphosis in NTK and THK groups.

**Table 1:** Demographic Characteristics are shown for all included adolescent idiopathic scoliosis (AIS) and controls.

	AIS				Controls
Included	Pre-operation	Immediate Post-operation	1-year Follow-up	2-year Follow-up	
No. of subject	27	27	27	20	36
Age (years)	18.1 ± 4.6	18.2 ± 4.6	19.5 ± 4.7	21.9 ± 4.9	25.1 ± 2.7
Cobb angle (°)	65.4 ± 11.9	21.5 ± 8.2	22.0 ± 9.0	22.6 ± 8.2	–
Correction rate (%)	–	67.7 ± 9.9	67.0 ± 10.5	65.2 ± 10.8	–
Loss of correction (°)	–	–	0.6 ± 9.7	− 0.3 ± 13.0	–
Normal Thoracic Kyphosis^#^	20	20	20	16	–
T5-T12 kyphosis (°)	23.6 ± 7.9	–	–	–	
Age (years)	18.7 ± 5.0	18.8 ± 5.1	20.2 ± 5.2	22.5 ± 5.0	
Cobb angle (°)	66.3 ± 13.3	22.1 ± 8.8	23.1 ± 9.9	22.6 ± 9.2	–
Correction rate (%)	–	67.2 ± 10.5	65.7 ± 11.3	64.7 ± 11.7	–
Loss of correction (%)	–	–	1.4 ± 10.9	1.3 ± 13.4	–
Thoracic Hypokyphosis^#^	7	7	7	3	–
T5-T12 kyphosis (°)	1.7 ± 6.6	–	–	–	
Age (years)	16.4 ± 3.1	16.5 ± 3.1	17.8 ± 2.8	17.1 ± 0.3	
Cobb angle (°)	62.9 ± 7.6	19.6 ± 6.7	18.9 ± 5.7	22.6 ± 2.1	–
Correction rate (%)	–	69.4 ± 7.7	70.3 ± 6.6	67.9 ± 2.6	–
Loss of correction (%)	–	–	− 1.6 ± 4.6	− 9.2 ± 3.6	–

Data expressed as mean ± SD.

^#^The values are based on the number of subjects, with the percentage in parentheses.

Intraclass correlation coefficient values for the intra- and inter-observer agreement of sagittal parameters, such as CL, SVA/SFD and pelvic obliquity, were higher than 0.9, indicating reliable measurement methods.

### Spinal alignment

**Figure 2:**
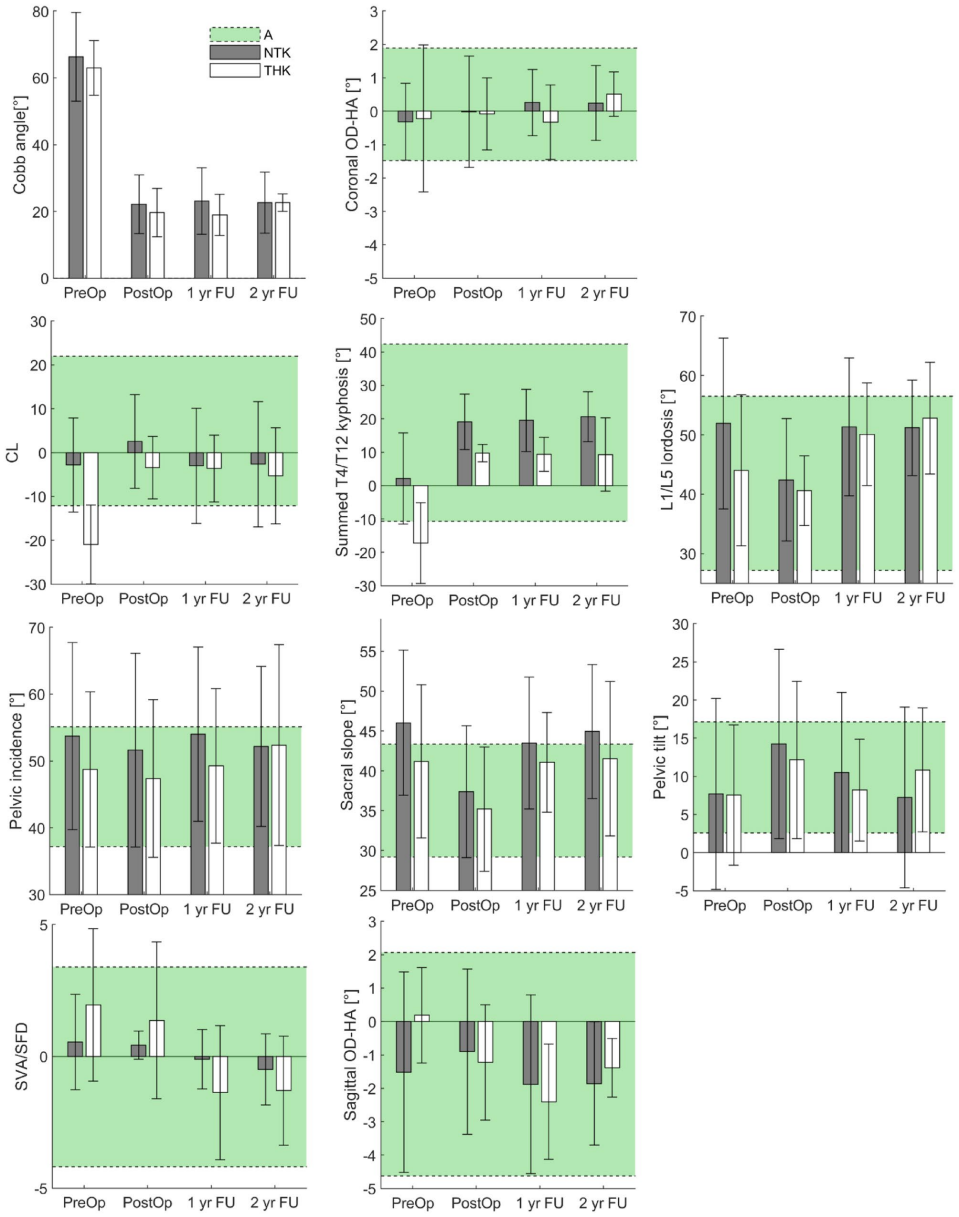
Main results comparing asymptomatic subjects (A, green shaded areas representing average ± 2*standard deviation), normal thoracic kyphosis patients (NTK, mean ± 2SD) and thoracic hypokyphosis patients (THK).

Figure 2 shows the main results and group comparisons. In AIS with THK, there was significant change in CL and TK from pre-operatively to each post-operative stage (*P* < 0.05), while no significant change was found in LL at any stage (*P* > 0.05) (Table 2). Pre-operatively, THK patients showed significant differences in CL and TK when compared with the asymptomatic controls (*P* < 0.05). TK significantly improved immediately after surgery (*P* < 0.05).

**Table 2:** Spinopelvic and global sagittal alignment parameters pre-operatively, immediate post-operatively, 1-year and 2-year follow-up AIS subjects and controls.

		Pre-operation	Immediate Post-operation	1-year Follow-up	2-year Follow-up	
**Spinal parameters**						
Cobb angle (°)	NC	–	–	–	–	
	NTK-AIS	66.3 ± 13.3	22.1 ± 8.8*	23.1 ± 9.9*	22.6 ± 9.2*	
	THK-AIS	62.9 ± 7.6	19.6 ± 6.7*	18.9 ± 5.7*	22.6 ± 2.1*	
Cervical lordosis (C2-C7) (°)	NC	4.9 ± 8.5	–	–	–	
	NTK-AIS	− 2.8 ± 10.7^#^	2.6 ± 10.7	− 3.0 ± 13.1^#^	− 2.7 ± 14.3	
	THK-AIS	− 20.9 ± 8.3^#^	− 3.4 ± 6.6*^#^	− 3.6 ± 7.1*^#^	− 5.3 ± 9.0	
Thoracic kyphosis (T4-T12) (°)	NC	15.8 ± 13.3	–	–	–	
	NTK-AIS	2.2 ± 13.6^#^	19.1 ± 8.3*	19.5 ± 9.3*	20.7 ± 7.5*	
	THK-AIS	− 17.3 ± 11.2^#^	9.7 ± 2.4*^#^	9.4 ± 4.7*	9.3 ± 9.0*	
Lumbar lordosis (L1-L5) (°)	NC	41.8 ± 7.3	–	–	–	
	NTK-AIS	51.9 ± 14.4^#^	42.4 ± 10.3	51.3 ± 11.6^#^	51.2 ± 8.1^#^	
	THK-AIS	44.0 ± 11.4	40.6 ± 5.2	50.1 ± 7.7	52.8 ± 6.7	
**Spino-pelvic parameters**						
Pelvic incidence (PI) (°)	NC	46.1 ± 9.0	–	–	–	
	NTK-AIS	54.8 ± 12.8^#^	52.3 ± 13.6^#^	54.6 ± 12.2^#^	53.2 ± 12.0^#^	
	THK-AIS	50.1 ± 14.4	49.3 ± 14.2	50.0 ± 14.4	55.3 ± 17.6	
Pelvic tilt (PT) (°)	NC	9.9 ± 7.3	–	–	–	
	NTK-AIS	9.6 ± 12.0	16.3 ± 11.7*^#^	11.5 ± 9.7	9.1 ± 11.3	
	THK-AIS	5.0 ± 9.4	9.5 ± 11.2	6.9 ± 8.0	9.7 ± 11.3	
Sacral slope (SS) (°)	NC	36.3 ± 7.1	–	–	–	
	NTK-AIS	45.2 ± 9.2^#^	36.1 ± 7.9*	43.1 ± 7.3^#^	44.2 ± 8.9^#^	
	THK-AIS	45.1 ± 10.3^#^	39.8 ± 6.7	43.1 ± 7.0^#^	45.7 ± 8.1	
Pelvic obliquity (PO) (°)	NC	0.8 ± 0.6	–	–	–	
	NTK-AIS	1.8 ± 0.8^#^	1.3 ± 0.6*^#^	1.4 ± 0.8^#^	1.3 ± 0.6*^#^	
	THK-AIS	1.6 ± 0.4^#^	1.3 ± 0.8*	1.3 ± 0.4	1.3 ± 0.2	
**Global sagittal alignment parameters**						
SVA (mm)	NC	− 2.6 ± 16.0	–	–	–	
	NTK-AIS	− 0.4 ± 22.5	− 14.4 ± 20.3*^#^	6.1 ± 22.4	− 6.1 ± 22.8	
	THK-AIS	− 8.0 ± 13.0	− 1.3 ± 17.0	11.5 ± 13.0	− 5.4 ± 15.8	
SFD (mm)	NC	− 28.4 ± 15.1	–	–	–	
	NTK-AIS	− 21.8 ± 20.8	− 36.4 ± 19.0*	− 27.1 ± 18.8	− 18.7 ± 28.2	
	THK-AIS	− 17.0 ± 15.9	− 24.2 ± 20.2	− 23.4 ± 13.9	− 26.3 ± 17.9	
SVA/SFD	NC	− 0.4 ± 1.9	–	–	–	
	NTK-AIS	0.5 ± 1.8	0.4 ± 0.5	− 0.1 ± 1.1	− 0.5 ± 1.3	
	THK-AIS	2.0 ± 2.7^#^	1.4 ± 2.8	− 1.4 ± 2.4*	− 1.3 ± 1.7	
Sagittal OD-HA (°)	NC	0.2 ± 0.8	–	–	–	
	NTK-AIS	− 0.3 ± 1.1	0.0 ± 1.7	0.3 ± 1.0	0.2 ± 1.1	
	THK-AIS	− 0.2 ± 2.2	− 0.1 ± 1.1	− 0.3 ± 1.1	0.5 ± 0.7	
Coronal OD-HA (°)	NC	− 1.3 ± 1.6	–	–	–	
	NTK-AIS	− 1.5 ± 3.0	− 0.9 ± 2.5	− 1.9 ± 2.7	− 1.9 ± 1.8	
	THK-AIS	0.2 ± 1.3^#^	− 1.2 ± 1.6	− 2.4 ± 1.6*	− 1.4 ± 0.9	

*NC* asymptomatic controls;* NTK* normal thoracic kyphosis;* THK* thoracic hypokyphosis;* SVA* sagittal vertical axis;* SFD*, sacro-femoral distance;* OD-HA*, odontoid-hip axis angle.

Data expressed as mean ± SD.

*P < 0.05, when compared with AIS at pre-operatively.

^#^ *P* < 0.05, when compared with NC.

TK significantly improved post-operatively in NTK patients as well (*P* < 0.05), with values similar to asymptomatic subjects (*P* > 0.05), and remained thus during follow-up. The change of TK was correlated with the change of LL (R = 0.498, *P* < 0.05) (Table 3). Although CL also improved at immediate post-operatively in older patients (age > 18, *P* = 0.04), there was no change in younger patients (*P* = 0.4) and all patients underwent a regression of CL at 1-year follow-up.

**Table 3:** Correlation between spinopelvic and global sagittal alignment parameters pre-op AIS with normal thoracic kyphosis (NTK).

NTK	CA	CL	TK	LL	PI	PT	SS	PO	SVA	SFD	SVA/SFD	Sag OD-HA	Cor OD-HA
CA	–	N.S	N.S	N.S	N.S	N.S	N.S	.549*	− .447*	N.S	N.S	N.S	N.S
CL		–	N.S	N.S	N.S	N.S	N.S	N.S	N.S	N.S	N.S	N.S	N.S
TK			–	0.498*	N.S	N.S	N.S	N.S	N.S	N.S	N.S	N.S	N.S
LL				–	N.S	N.S	.691**	N.S	N.S	N.S	N.S	N.S	N.S
PI					–	.711**	N.S	N.S	N.S	− .738**	N.S	N.S	N.S
PT						–	N.S	N.S	N.S	− .946**	N.S	N.S	N.S
SS							–	N.S	N.S	N.S	N.S	N.S	N.S
PO								–	N.S	N.S	N.S	N.S	N.S
SVA									–	N.S	− .464*	− .563*	N.S
SFD										–	.527*	.522*	N.S
SVA/SFD											–	.796**	N.S
Sag OD-HA												–	N.S
Cor OD-HA													–

Significance level: *P < 0.05, **P < 0.01.

*NTK* normal thoracic kyphosis; *CA* Cobb angle; *CL* cervical lordosis; *TK* summed thoracic kyphosis; *LL* lumbar lordosis; *PI* pelvic incidence; *PT* pelvic tilt; *SS* sacral slope; *PO* pelvic obliquity; *SVA* sagittal vertical axis; *SFD* sarco-femoral distance; *Sag OD-HA* sagittal odontoid-hip axis angle; *Cor OD-HA* coronal odontoid-hip axis angle.

### Spinopelvic alignment

In AIS with THK, no significant change was found in PI, PT and SS at any stage, independently of age (*P* ≥ 0.05). Although significant changes of pelvic obliquity were found at immediate post-operative, these parameters were normalized at 1-year follow-up (*P* > 0.05). When compared with asymptomatic controls, there was significant difference in SS and pelvic obliquity pre-operatively and 1-year follow-up in THK group (*P* < 0.05). Also, the changes in LL showed a positive correlation with SS (R = 1.000, *P* < 0.01) (Table 4).

**Table 4:** Correlation between spinopelvic and global sagittal alignment parameters pre-op AIS with thoracic hypokyphosis (THK).

THK	CA	CL	TK	LL	PI	PT	SS	PO	SVA	SFD	SVA/SFD	Sag OD-HA	Cor OD-HA
CA	–	N.S	N.S	N.S	N.S	N.S	N.S	N.S	N.S	N.S	N.S	N.S	N.S
CL		–	N.S	N.S	N.S	N.S	N.S	N.S	− .775*	N.S	N.S	N.S	N.S
TK			–	N.S	N.S	.857*	N.S	.929**	N.S	− .821*	N.S	N.S	N.S
LL				–	N.S	N.S	1.000**	N.S	N.S	N.S	N.S	− .975**	N.S
PI					–	.775*	N.S	N.S	N.S	N.S	N.S	− .827*	N.S
PT						–	N.S	N.S	N.S	− .964**	N.S	N.S	N.S
SS							–	N.S	N.S	N.S	N.S	N.S	N.S
PO								–	N.S	N.S	N.S	N.S	N.S
SVA									–	N.S	N.S	N.S	N.S
SFD										–	N.S	N.S	N.S
SVA/SFD											–	N.S	N.S
Sag OD-HA												–	N.S
Cor OD-HA													–

Significance level: *P < 0.05, **P < 0.01.

*THK* thoracic hypokyphosis; *CA* Cobb angle; *CL* cervical lordosis; *TK* summed thoracic kyphosis; *LL* lumbar lordosis; *PI* pelvic incidence; *PT* pelvic tilt; *SS* sacral slope; *PO* pelvic obliquity; *SVA* sagittal vertical axis; *SFD* sarco-femoral distance; *Sag OD-HA* sagittal odontoid-hip axis angle; Cor OD-HA, coronal odontoid-hip axis angle.

In NTK group, significant changes were found in PT, SS and pelvic obliquity between pre-operatively and immediate post-operatively, independently of age (*P* < 0.05), although the change in PT was at the limit of significance in young adults (*P* = 0.05), and all these changes were normalized to pre-operative values at 1-year follow-up (*P* > 0.05). PI did not change in average (*P* > 0.05), but 7 patients (35%) showed changes of 5° or more in 1-year follow-up. In addition, there were significant differences in PI, SS and pelvic obliquity between AIS patients pre-operatively and at 1-year follow-up when compared with asymptomatic controls. And similar to AIS with THK, the increase in PI was correlated to the change in PT (R = 0.711, *P* < 0.01) (Table 3).

### Head and global sagittal alignment

Although there was no significant change in SVA, SFD and coronal OD-HA in AIS with THK, independently of age, there was a significant decrease in the ratio of SVA/SFD at 1-year follow-up (*P* < 0.05) and a decrease of sagittal OD-HA (*P* < 0.05). Both SVA/SFD and sagittal OD-HA were significantly different between AIS patients at pre-operation and asymptomatic controls (*P* < 0.05). After surgery, these parameters improved towards the values of the asymptomatic subjects (Fig. 2).

For AIS patients with NTK, there were significant changes of SVA and SFD immediate post-operatively (*P* = 0.01). However, these changes lost significance when looked at by age group (*P* ≥ 0.05), and they returned to pre-operative values when follow-up after 1-year and 2-year, when they remained similar to controls at all time points (*P* > 0.05). No significant correlation in SVA with these parameters was found, while SFD was correlated to the change in PI (R =  − 0.738, *P* < 0.01) and PT (R =  − 0.946, *P* < 0.01). And unlike AIS patients with THK, there was no significant correlation with the ratio of SVA/SFD and sagittal parameters being measured. However, there was correlation found between sagittal OD-HA and the change in SVA (R =  − 0.563, *P* < 0.05) and SFD (R = 0.522, *P* < 0.05). OD-HA was also positively correlated with the ratio of SVA/SFD (R = 0.796, *P* < 0.01).

### Discussion and conclusion

In this study, a thorough analysis of global sagittal alignment from head to pelvis was performed in AIS patients with normal kyphosis and hypokyphosis, before and at different time-points after surgery. Several important results were highlighted. First, severe scoliosis patients were able to keep their coronal and sagittal balance (OD-HA within the corridor of normality, Fig. 2), but their sagittal balance can improve postoperatively. THK patients can compensate their particularly low kyphosis with a slightly lower lumbar lordosis, but also with a largely negative cervical lordosis, which allows them to keep the head upon the pelvis. Still, this compensation appears barely sufficient because their sagittal OD-HA was slightly positive. Second, spinopelvic parameters changed between the immediate postop and 1-year post-op. This confirms that sagittal alignment can change for a long time after surgery^11^, and that the short-term follow-up radiograph shows the patient while he is still adapting his compensation mechanisms to keep balance after surgery. For instance, although the position of the head of THK patients was normalized postoperatively (sagittal ODHA, Fig. 2), the SVA/SFD ratio was still high and became negative at 1-year follow-up, suggesting long-term adaptations of the balance chain. The third point is that the pelvis appeared to play a minor role in these compensation strategies, because average PT only changed in the immediate post-op of NTK patients.

The importance of global sagittal alignment, including the entire spine and pelvis, has been well-documented in the adult spine^12,13^. Duval-Beaupere et al*.* suggested that maintaining a human body in an upright position should require the least amount of energy to be balanced in terms of muscle fatigue and vertebral strain^5^. A disturbance on the sagittal balance can cause pain^14,15^ and, it can potentially result from the progression of spinal curvature into adulthood. Some studies suggested an association between coronal deformity and sagittal deformity^16,17^. including an increase of PI in AIS patients^3^. Despite the differences, these studies utilized a heterogeneous AIS group with different curve types. Thus, the current study minimized such concern by recruiting a homogenous group of AIS subjects with right-sided thoracic curvature only. Based on our findings, significant different sagittal profile adaptation was found between AIS with and without THK.

**Figure 3:**
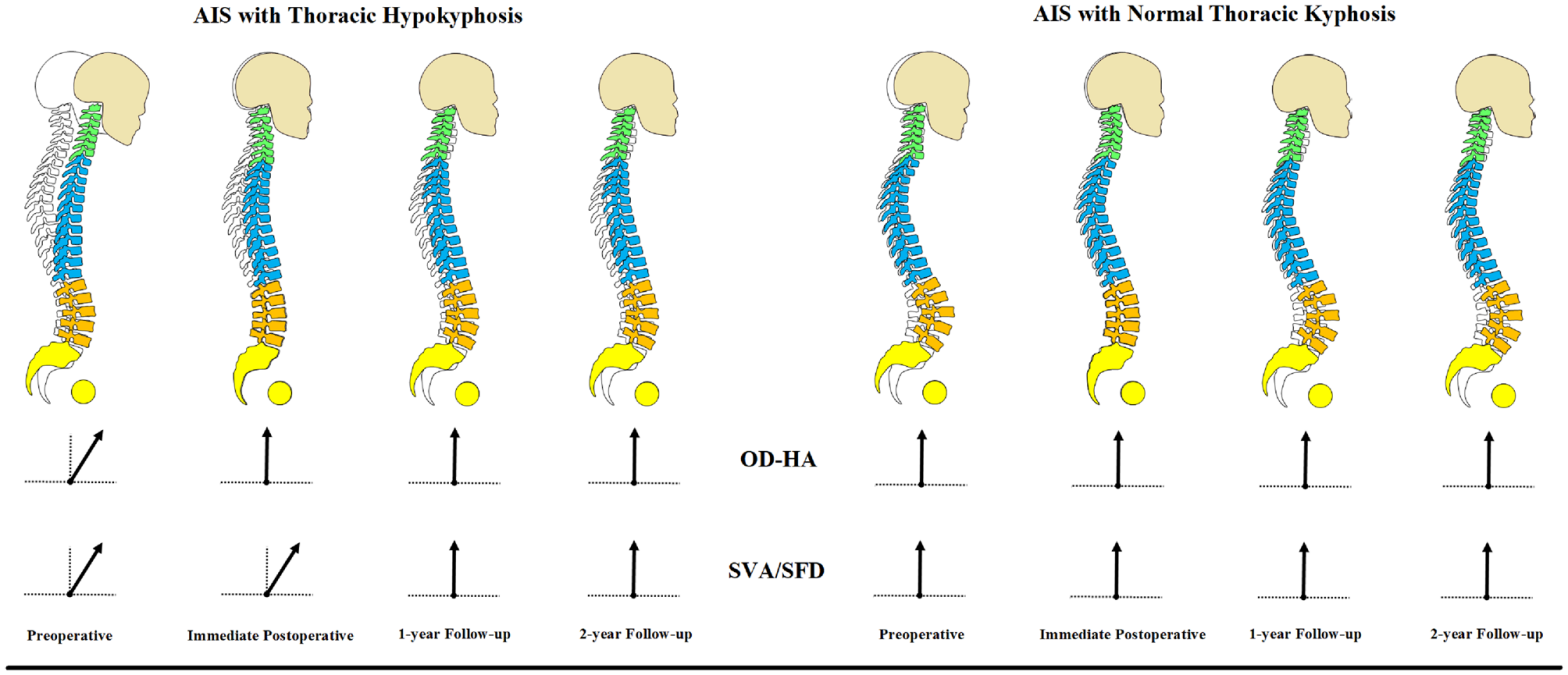
The schematic diagram on the evolution of changes in the global sagittal profiles of AIS patients with and without thoracic hypokyphosis pre-operative and at different time points post-operative. In AIS patients with thoracic hypokyphosis, the overall balance is compensated with the unusually forward leaning posture (head forward) in pre-operative stage. This posture is corrected with modification during different stages post-operatively. Whereas in AIS patients with normal thoracic kyphosis, the overall balance is maintained with head in a relatively neutral position. There is no significant interval change of the thoracic kyphosis at any time point. The spinal profiles are superimposed on a non-scoliotic spine. The arrow represents the overall balance of the corresponding parameters at each time point: ↗, leaning forward; ↑, medial balanced.

In AIS patients with THK, the overall balance was compensated with an unusual forward leaning posture at pre-operative stage. This was accompanied by a reversed CL, TK and high SS, but normal LL. This posture was corrected with modification during different stages post-operatively, but not to normal values. This indicated that THK patients need to undergo substantial change of their sagittal profiles to find a new improved balance, including a posterior shift of the trunk, whereas a similar maneuver was not necessary for NTK patients. Moreover, this represents a further confirmation immediate post-op postural configuration is mostly transient, and it may not truly reflect long-term surgical results (Fig. 3). Part of these changes in sagittal alignment could be due to behavioral rather than physiological causes, such as increased use of electronics (tablets, smartphones, etc.) in youth^18,19^.

It was previously shown the sagittal alignment in cervical region can be influenced by the corrective spinal instrumentation^20^. The spinal fusion at thoracic region could reflect the reciprocal decrease in LL on patients with pedicle subtraction osteotomy^21^. Likewise, many studies showed that LL was significantly correlated with sacral slope^12,20^. Though PI remained relatively constant, the compensatory mechanism induced a simultaneous increase in PT and a decrease in SS before and after surgery^12,22,23^.

Proximal junctional kyphosis is a recent recognized phenomenon in AIS to affect global sagittal alignment after corrective surgery^24^. Hence, neglecting the position of the head during alignment assessment might result in an incomplete evaluation of overall balance pre-operatively for the outcome after corrective spinal surgery. Our previous studies demonstrated the OD-HA angle has little variation among the population^8,9,25^. Indeed, this would remain within a normal range even in surgically-operated AIS patients who have developed proximal junctional kyphosis^13^. The more commonly used parameter to evaluate the global sagittal alignment is the SVA/SFD ratio^26^. The ratio of SVA/SFD was smaller in both groups during immediate post-operative follow-up, which indicated that the balance was only partially compensated. On the other hand, OD-HA was only abnormal in AIS with THK prior to surgical correction. This normalization might suggest that the surgery have helped THK patients to achieve a compensatory balance immediate after correction, which the ratio of SVA/SFD failed to indicate. While for the NTK group, the OD-HA remained within normal range at all time-points, suggesting normal balance (Fig. 3).

The possible explanation of the above observations is that the head plays a vital role in adjustment of balance during immediate and delayed post-operative periods. AIS patients with instrumental spine need to adopt a new sagittal profile before the natural physiological balance can be achieved. The normalization of OD-HA soon after post-op in THK patients suggests that a reasonably compensated balance was achieved. Yet, close monitoring is still required for these patients as degenerative changes of cervical region and symptomatic pain may occur, with progression to adulthood^17^.

Although the sample size for this study was relatively small for a generalization of the results, especially in the THK cohort, it still serves as a reference to the significant changes observed on the spinopelvic parameters, and as a guideline for future investigations, which should systematically include the analysis of the position of the head. The mean age of the control group was slightly higher than the AIS group, and the AIS cohort included patients over 21 years old at the moment of surgery; this is due to the long waiting times for non-life-threatening surgeries at the at our center—one of the only two the public tertiary hospital designated for AIS surgery. All AIS patients in this study presented with a Risser sign of 4–5 indicating a cessation of skeletal growth. Thus, there should be no significant age-effect on the sagittal profile between patients and controls. Also, with only the position of the head being included in the analysis, the relation with the lower limbs remains unknown. Hence, incorporating the global sagittal alignment imaging protocol to include the head and lower limbs would be recommended. In addition, a functional posture balance examination, e.g. gait analysis, would help to better elucidate these changes before and after surgery.

In conclusion, this study found the changes in global sagittal alignment and mechanism of balance are different in AIS with or without THK. The measurement of OD-HA may be complimentary parameter for global balance assessment. However, further validation with more expanded studies in using larger cohorts across multiple centers might help to incorporate this finding as a useful post-operative assessment and follow-up for AIS patients.

## Materials and methods

### Subject recruitments

Chinese female adolescents and young adults with AIS, which was confirmed clinically and radiologically, were prospectively recruited at our scoliotic clinics between June 2015 and Feb 2017. Inclusion criteria were: (1) a major right thoracic curvature with Cobb angle higher than 45°, (2) a planned surgical treatment by posterior instrumentation with pedicle screw rod construct and fusion with a lower instrumented vertebra above or at L2, and (3) an absence of pre-operative systemic disorder or neurologic deficit. Patients with secondary scoliosis of known etiology, such as neuromuscular scoliosis and congenital scoliosis, were excluded. Asymptomatic girls were recruited from local schools and screened by experienced orthopedic surgeons to exclude the presence of scoliosis. Other demographic variables, such as the age when the radiographs were made, were also collected.

All study procedures were approved by the institutional review board equivalent ethical committee in our institution (Joint Chinese University of Hong Kong-New Territories East Cluster Clinical Research Ethics Committee, reference 2016.722) and conducted in accordance to the Declaration of Helsinki. Written informed consent was obtained from the subjects or their parents prior to participating in this study.

### Radiological evaluation

**Figure 4:**
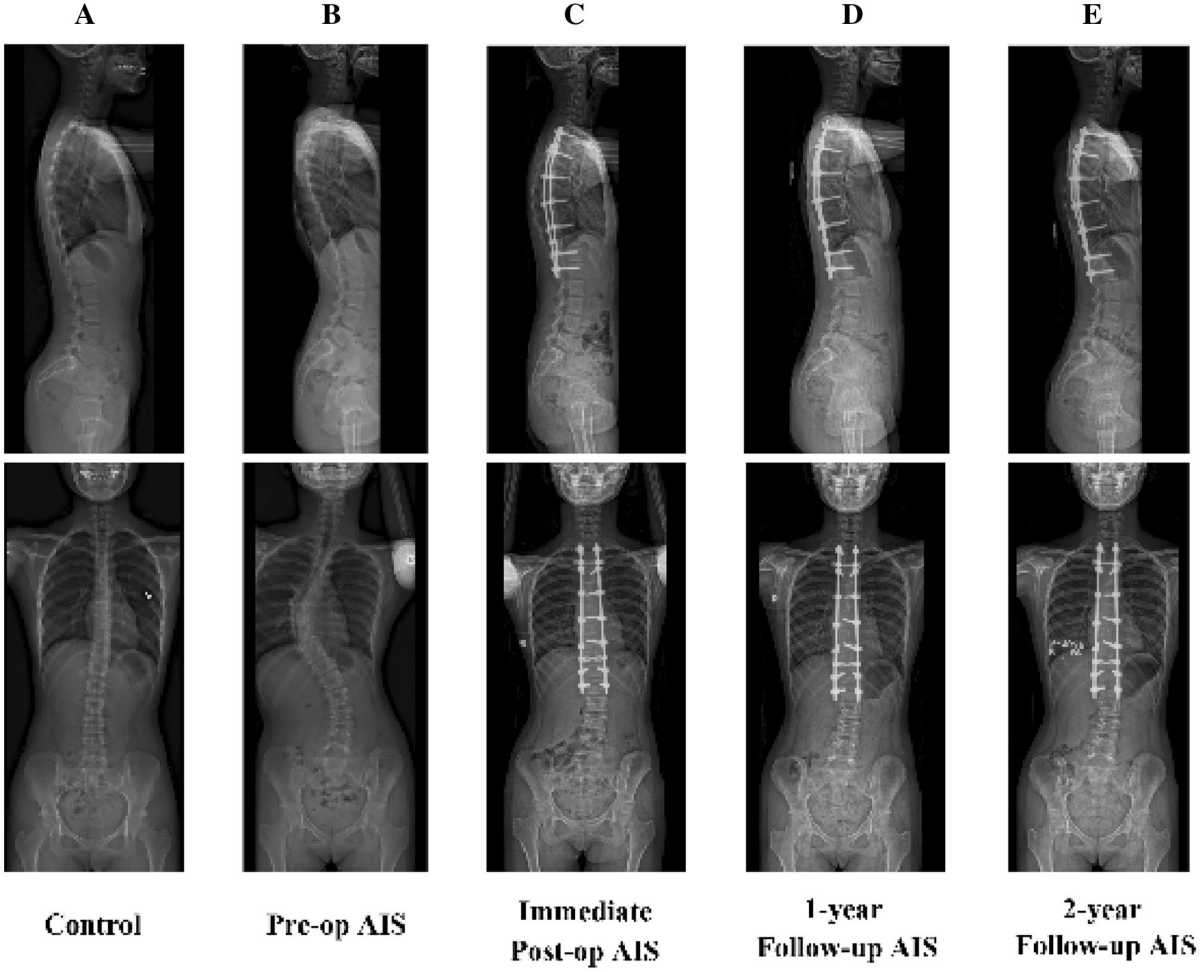
Sagittal radiographs of an asymptomatic female control (**A**) and a female patient with adolescent idiopathic scoliosis taken pre-operatively (**B**), and immediate post-operatively (**C**), at 1-year (**D**) and 2-year (**E**) follow-up.

For each subject, biplanar radiographs (EOS imaging, Paris, France) were acquired with a standardized radiographic protocol in standing position^13^. Acquisitions were performed pre-operatively, at immediate post-operatively (1–3 months), 1-year and 2-year follow-up^27^ (Fig. 4). Patients not in free-standing position were excluded.

**Figure 5:**
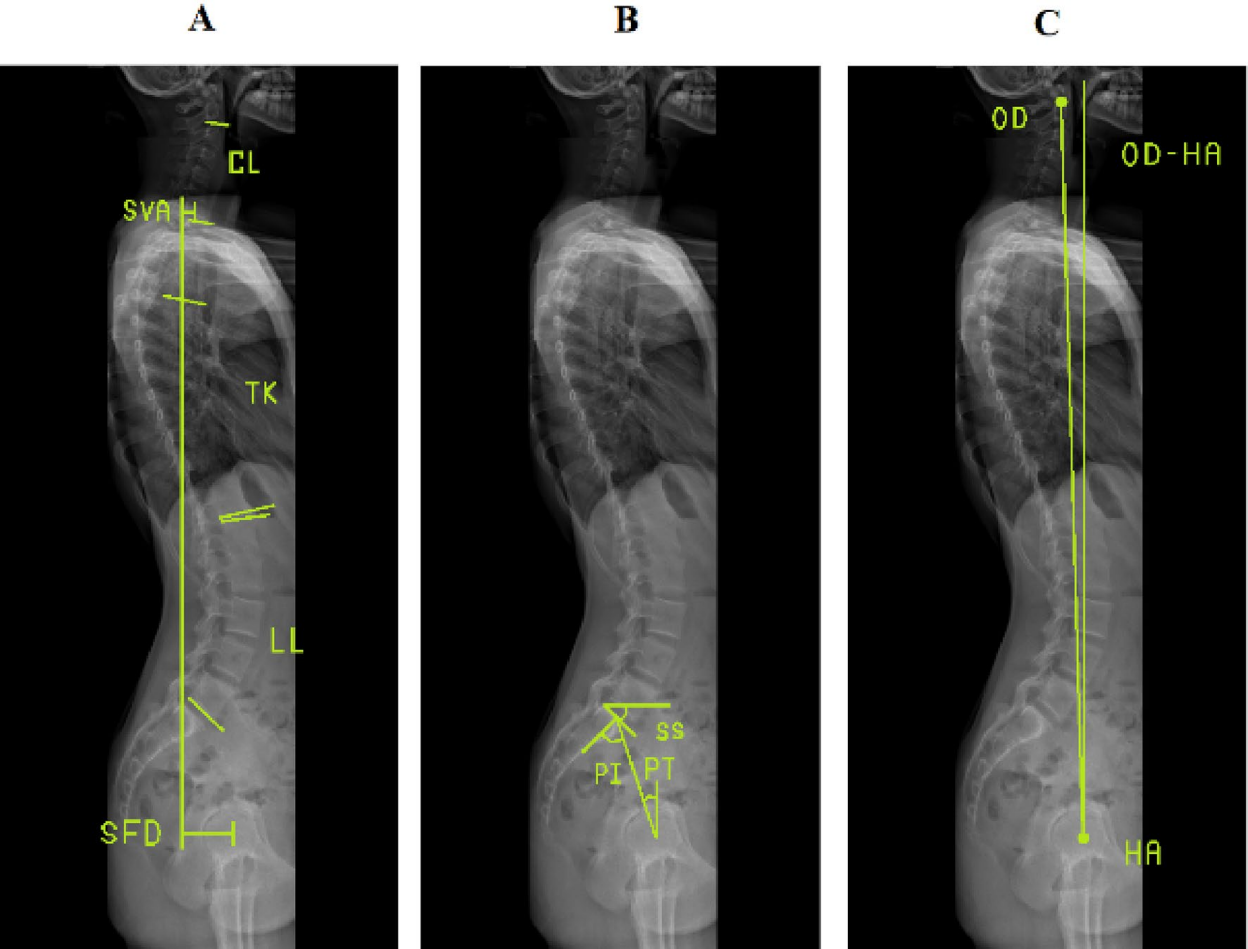
Illustration of measurements of spinal, pelvic and OD-HA parameters in sagittal radiography, including (**A**) *CL* cervical lordosis; *TK* thoracic kyphosis; *LL* lumbar lordosis; *SVA* sagittal vertical axis; *SFD* sacro-femoral distance; (**B**) *PI* pelvic incidence; *SS* sacral slope; *PT* pelvic tilt; and (**C**) OD-HA, odontoid-hip axis angle.

A 3-D patient-specific model was built (SterEOS software, Paris, France), which allowed the automatic computation of Cobb angle and multiple sagittal radiographic parameters, including cervical lordosis (CL), lumbar lordosis (LL), sagittal vertical axis (SVA), sacro-femoral distance (SFD), as well as the ratio between the C7 plumb line from the postero-superior corner of the sacrum and SFD (SVA/SFD) (Fig. 5A). Standard spinopelvic parameters were also computed: pelvic incidence (PI), pelvic tilt (PT), sacral slope (SS), and pelvic obliquity (Fig. 5B).

In addition, 3-D segmental summed kyphosis was also calculated, using the method described by Newton et al*.*^28^. In brief, local vertebral kyphosis was calculated as the angle between the vectors normal to the two endplates, which was projected on the vertebral sagittal plane. The same approach was used to calculate disc kyphosis, using the adjacent vertebral endplates. Finally, T4-T12 summed kyphosis was obtained by summing the local kyphosis of all vertebrae and disc between the T4 and T12. Kyphosis was noted as a positive value; lordosis was recorded as a negative value, and clockwise rotation was defined as positive.

### 3-D measurements of odontoid-hip axis angle

The position of the most superior point of dentiform apophyse of C2 (OD) was obtained using a validated technique^29^. The odontoid-hip axis angle (OD-HA) was defined as the angle between the vertical line crossing the center of the hip axis (HA) and a line between OD and HA; this angle was computed both in sagittal and coronal views (Fig. 5C).

### Statistical analyses

The power analysis on the sample size was performed using G*Power (version 3.1.9.1, HHU)^30^: assuming that patients would show a normal OD-HA parameter postoperatively (− 2.3 ± 2.0° according to Amabile et al.^8^), and aiming to detect an average change of 1°, a THK cohort of 7 patients yielded a 1-β power of 0.85. The size of the NTK cohort was the result of including enough patient to attain the minimal cohort of THK patients. All other statistical analysis was performed with a commercial software (SPSS software version 25.0; IBM SPSS). Normal distribution of the values was confirmed by Shapiro–Wilk normality test for each series of measurements. For data with normal distribution, analysis of variance with Bonferroni correction was used for comparison. Comparison between pre- and post-op parameters with non-normal distribution was performed using paired Wilcoxon signed rank test while comparison with healthy control using Mann Whitney U test. Sub-analyses were performed by age group to compare younger patients (age ≤ 18 years, N = 17) with young adults (age ≥ 21, N = 10). Correlations between spinopelvic parameters were analyzed using Spearman correlation test. The inter-observer and intra-observer reliability were assessed with an absolute agreement intraclass correlation coefficient analysis using a two-way random effects model. Agreement was classified as excellent for an intraclass correlation coefficient of > 0.75. Values of *P* < 0.05 was considered statistically significant.

### Ethics approval and consent to participate

The study procedure was conducted in accordance to guidelines approved by the institutional clinical research ethics committee (CREC No. 2016.722) and the Declaration of Helsinki. Written informed consent was obtained from all subjects and their parents before participating in this study.

### Consent for publication

All authors have given permission for publication. Consent for publication of the subject not applicable.

## Data Availability

The datasets used in this manuscript and all analyzed data from this study are available from the corresponding author upon reasonable request.

## Abbreviations

AIS: Adolescent idiopathic scoliosis
CL: Cervical lordosis
LL: Lumbar lordosis
NTK: Normal thoracic kyphosis
OD-HA: Odontoid-hip axis angle
PI: Pelvic incidence
PT: Pelvic tilt
SFD: Sacro-femoral distance
SS: Sacral slope
SVA: Sagittal vertical axis
SVA/SFD: Ratio between the C7 plumb line from the postero-superior corner of the sacrum and SFD
THK: Thoracic hypokyphosis
TK: Thoracic kyphosis
